# Development of coronary artery lesions in indolent kawasaki disease following initial spontaneous defervescence: a retrospective cohort study

**DOI:** 10.1186/s12969-015-0042-8

**Published:** 2015-11-04

**Authors:** Takuto Takahashi, Hiroshi Sakakibara, Yoshihiko Morikawa, Masaru Miura

**Affiliations:** Department of General Pediatrics, Tokyo Metropolitan Children’s Medical Center, 2-8-29 Musashidai, Fuchu, Tokyo 183-8561 Japan; Clinical Research Support Center, Tokyo Metropolitan Children’s Medical Center, 2-8-29 Musashidai, Fuchu, Tokyo 183-8561 Japan; Department of Cardiology, Clinical Research Support Center, Tokyo Metropolitan Children’s Medical Center, 2-8-29 Musashidai, Fuchu, Tokyo 183-8561 Japan

**Keywords:** Immunoglobulin, Defervesced, Self-limiting, Afebrile

## Abstract

**Background:**

No standard treatment protocol exists for Kawasaki disease (KD) after spontaneous defervescence that does not use intravenous immunoglobulin (IVIG). Moreover, some cases present an indolent course and later develop coronary artery lesions (CALs). We aimed herein to assess the short-term prognosis of KD after defervescence and to clarify the characteristics of indolent KD.

**Study design:**

The present report is the culmination of a 10-year retrospective cohort study of KD at two Japanese tertiary pediatric centers. Cases of spontaneously defervesced KD ≤ 7 days of illness without IVIG which were afebrile for ≥ 3 days were classified as ‘defervesced KD’ (dKD). Of these, cases which developed CALs, or received IVIG for ongoing systemic inflammation were sub-classified into the indolent KD group (iKD). The primary outcome was the prevalence of CALs.

**Results:**

Among 968 KD patients, 7.3 % (71/968) fell into the dKD, and 11.2 % (8/71) into the iKD, groups. No difference in CAL prevalence was observed between the dKD group and the KD group treated with IVIG (9.9 % vs. 7.6 %, p value = 0.49). Six of the 8 iKD cases developed CALs between Days 11 and 23 (median 16) of illness. All iKD cases presented recurrent fever or re-elevated CRP; however, there were generally fewer persistent KD symptoms than at diagnosis.

**Conclusions:**

The prognosis of spontaneously defervesced KD was relatively benign. However, indolent cases with ongoing, systemic inflammation developed CALs. In some cases, immediate IVIG use can be withheld as long as recurrent fever and re-elevation of CRP are monitored and supplementary echocardiogram are conducted.

## Background

Kawasaki disease (KD) is an acute, self-limiting, childhood vasculitis marked by a constellation of fever and various acute inflammatory features. Although these clinical symptoms resolve in on average 11 days without treatment, coronary artery lesions (CALs) may later develop [1]. Prompt intravenous immunoglobulin (IVIG) therapy within 7–10 days of illness is the current, internationally recognized method of managing KD with the specific aim of preventing CALs [1, 2]. However, the timely commencement of IVIG therapy is hampered by the lack of definitive diagnostic tests and by the theoretical risk of blood-borne infection accompanying IVIG use [3], the cost of treatment, and dependence on blood donations. As a result, some KD patients experience spontaneous defervescence before IVIG administration.

The optimal management of KD in patients who experience spontaneous defervescence remains uncertain. For this population, the Japanese KD management guidelines recommend that the decision to withhold IVIG should be made after the laboratory test and echocardiography results are considered [2]. While the American Heart Association (AHA) does not suggest withholding IVIG treatment in KD patients presenting ≤ 10 days of illness. For afebrile KD presenting >10 days of illness the AHA recommends that IVIG be administered, albeit only to cases presenting an aneurysm or ongoing systemic inflammation [1]. However, some atypical KD cases display an indolent clinical course presenting initial spontaneous remission of fever followed by the development of CALs [4]. To our knowledge, no previous study has specifically addressed this issue.

Our aims in this study were first, to assess the clinical outcomes in patients with spontaneously defervesced KD who did not initially undergo IVIG therapy; second, to define the term ‘indolent KD’ by describing the characteristics of patients in this subgroup; and finally, thereby to suggest an appropriate method of managing this condition.

## Methods

### Study design

The present study was designed as a retrospective cohort study using institutional databases for the national registry for KD at two tertiary children’s hospitals in Tokyo, Japan between January, 2005 and December, 2014. All patients enrolled in the study underwent a minimum follow-up period of 3 months with an echocardiogram in order to evaluate the CAL complication rate. This study was approved by the Ethical Committee of Tokyo Metropolitan Children’s Medical Center.

The patients were classified into the defervesced KD group (dKD) if they initially experienced spontaneous defervescence without receiving IVIG or glucocorticoid therapy within 7 days of illness and remained afebrile for more than 3 days. Otherwise they were placed into the treated KD group (tKD). Among the dKD group, those who developed CALs or received IVIG to treat suspected, residual inflammation during 10 or more days of illness were further classified into the indolent KD group (iKD). The rest of the dKD group was classified into the resolved KD group (rKD).

The primary outcome was the rate of CAL occurrence within 30 days of illness between the dKD, and the tKD, groups. The other variables included demographic data, duration of condition from initial hospital visit, rate of incomplete KD, duration of fever, number of fulfilled KD criteria, and laboratory findings. Day of illness was defined as the number of days from the first occurrence of fever. The latest laboratory findings in the febrile period were assessed. In addition, detailed characteristics of the dKD patients were assessed after the retrieval of information from the medical records of each patient. The AHA criteria for administering IVIG to incomplete KD cases were assessed for the dKD group [1].

### Definition

The national KD registry included both classic and incomplete KD as defined by the Japanese diagnostic guidelines for KD. The diagnosis of classic KD was made if at least 5 of the 6 criteria, namely, fever and the 5 principle clinical criteria for KD, were met. The diagnosis of incomplete KD was made in cases which strongly suggested KD but failed to fulfill the criteria for classic KD [5].

CALs were defined according to the Japanese Ministry of Health criteria as presenting a luminal diameter ≥ 3.0 mm in a child < 5 years old or ≥ 4.0 mm in a child ≥ 5 years old, or having either a segment with an internal diameter ≥ 1.5 times larger than that of an adjacent segment or a clearly irregular luminal contour [6]. Coronary aneurysms were sub-classified based on their internal diameter (ID) as: (1) a small aneurysm or a dilatation (ID ≤ 4 mm for < 5 years of age, and < 1.5 times that of the adjacent segment for ≥5 years of age), (2) a medium aneurysm (ID =4–8 mm for < 5 years of age, and 1.5–4.0 times larger than that of the adjacent segment for ≥5 years of age), or (3) a giant aneurysm (ID ≥ 8 mm for < 5 years of age, and > 4.0 times larger than that of the adjacent segment for ≥5 years of age) [7].

### Institutional protocols

All enrolled patients were treated according to the management guidelines for KD in Japan [2, 5]. Additional glucocorticoid administration in the initial therapy was provided according to the institutional protocols described below. Patients diagnosed with KD were treated with a single dose of IVIG (2 g/kg) delivered over 24 h and oral aspirin (30 mg/kg per day) during the acute stage. The dosage of aspirin was reduced (3–5 mg/kg per day) from the third day of the treatment except in IVIG-refractory cases. Patients who showed persistent or recurrent fever ≥ 24 h after completion of IVIG administration were re-treated with a second course of IVIG therapy. Intravenous methylprednisolone pulse therapy was administered as the third-line therapy to those who were unresponsive to the second course of IVIG [8].

Different types of additional, initial glucocorticoid therapy were administered in each of the 3 periods: in the first period (January, 2005 to September, 2008), glucocorticoids were not administered; in the second period (September, 2008 to December, 2010), prednisolone (2 mg/kg/day) was randomly added to the initial therapy and tapered over 2 weeks based on the RAISE study protocol [9]; and in the last period (December, 2010 to December, 2014), the same dosage of glucocorticoids was administered only to those who were at high risk of IVIG-refractory KD as defined by Kobayashi’s score [10].

In terms of the management of spontaneously defervesced KD, the 2012 revision of the Japanese guidelines for the treatment of KD recommends withholding IVIG for this population at the discretion of the treating physician after due consideration of the level of inflammatory markers and echocardiography results; the same approach was taken at our institutions before 2012. Although administration of IVIG in cases of incomplete KD was left to the discretion of the treating physicians, IVIG use was generally delayed in patients fulfilling fewer KD criteria or presenting lower levels of inflammatory markers.

A routine echocardiogram was performed for classic KD patients in the acute phase at the time of diagnosis, between days 5 and 9, at 1 month, and between months 2 and 3, after diagnosis.

### Data analysis

Fisher’s exact test was used for comparison of categorical variables and Student’s test or Mann Whitney test for comparison of continuous variables between the dKD and tKD groups, and between the iKD and rKD groups. A *p* value < 0.05 was considered to be statistically significant; there was no adjustment for multiple testing. All statistical analyses were carried out using the IBM SPSS statistics software for Windows 22.0.0 (IBM Corp, NY, USA).

## Results

**Fig 1:**
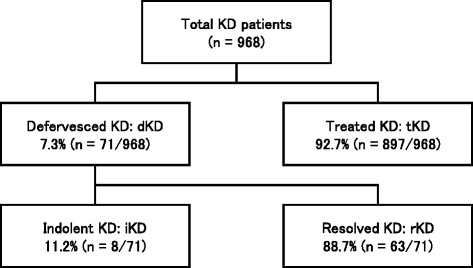
Study design diagram KD: Kawasaki disease

Registry data from a total of 968 KD cases were reviewed for the study. Among these, 71 (7.3 %) were cases of dKD, defined by spontaneous defervescence occurring within 7 days of illness and afebrility lasting ≥ 3 days without IVIG or glucocorticoid therapy. The remaining 897 cases (92.7 %) included only 1 patient who initially defervesced after receiving glucocorticoid monotherapy for an unknown inflammatory condition inferred from the presence of long-term fever. Of these 71 dKD cases, 8 (11.2 %) were iKD cases that developed CALs or received IVIG after ≥ 10 days of illness [Fig. 1].

A detailed review of patient records revealed the reasons why dKD cases were not treated with IVIG. Twenty-eight of these 71 dKD cases met the KD criteria. This group mainly included patients who met the diagnostic criteria of KD yet experienced spontaneous defervescence by the time of their initial hospital visit to our hospital. Induration of the BCG vaccination site was detected in 19 of the 43 dKD patients who did not meet the KD diagnostic criteria, strongly corroborating the diagnosis of incomplete KD. Only 2 of the 43 dKD patients with an incomplete KD diagnosis fulfilled the AHA criteria for IVIG administration.

The incidence of CAL and other variables in the dKD and tKD groups was compared (Table 1). There was no statistically significant difference in the CAL rate between these 2 groups (9.9 % vs. 7.6 %, *p* value = 0.49). The dKD group included a significantly higher number of incomplete KD diagnoses (59.2 % vs. 13.5 %, *p* value < 0.001).

**Table 1:** Comparison between the defervesced KD vs. treated KD groups

	Defervesced KD (*n* = 71)		Treated KD (*n* = 897)		*p* value
Age, months, < 12, n (%)	23	(32.4)	185	(20.6)	
12 to 60, n (%)	39	(54.9)	582	(64.9)	0.07
60 ≤, n (%)	9	(12.7)	130	(14.5)	
Total, mean (SD)	28	(22)	33	(26)	0.08
Male gender, n (%)	36	(50.7)	521	(58.1)	0.23
Days of illness at arrival, mean (SD)	4.5	(1.9)	4.2	(1.7)	0.20
Incomplete KD, n (%)	42	(59.2)	121	(13.5)	<0.001*
CAL, n (%)	7	(9.9)	68	(7.6)	0.49
Coronary aneurysm, n (%)					
Small	4	(5.6)	36	(4.0)	0.51
Medium	2	(2.9)	26	(2.9)	0.98
Giant	1	(1.4)	9	(1.0)	0.75

*KD* Kawasaki disease, *CAL* coronary artery lesion, *SD* standard deviation, *statistically significant defined as *p* <0.05

The comparison between the iKD and the rKD groups is shown in Table 2. In the iKD, fewer cases fulfilled the KD criteria than in the rKD group, and more cases exhibited lower hemoglobin, lower hematocrit, lower albumin, and higher CRP values.

**Table 2:** Comparison between the indolent KD vs. resolved KD groups

	Indolent KD (*n* = 8)		Resolved KD (*n* = 63)		*p* value
Age, months; mean (SD)	16.3	(15.9)	28.4	(23.1)	0.16
Male, n (%)	4.0	(50)	32.0	(50.8)	0.97
Incomplete KD, n (%)	6.0	(75)	36.0	(57.1)	0.33
Duration of fever, days; mean (SD)	4.6	(1.2)	4.7	(1.3)	0.93
No. of KD criteria, mean (SD)	3.0	(0.9)	3.8	(0.9)	0.04*
WBC, × 10^3^/μL; mean (SD)	12.1	(3.4)	12.5	(4.7)	0.81
Neutrophils, %; mean (SD)	53.5	(13.3)	54.6	(16.6)	0.86
Hb, g/L; mean (SD)	10.4	(0.9)	11.6	(1.2)	0.01*
Hct, %; mean (SD)	31.4	(3.0)	34.5	(2.7)	0.006*
Platelet, × 10^4^/μL; mean (SD)	44.6	(23.8)	35.2	(16.8)	0.18
T-bil, mg/L; mean (SD)	0.7	(3.7)	0.4	(1.3)	0.48
AST, IU/L; mean (SD)	26.1	(10.9)	47.9	(47.4)	0.23
ALT, IU/L; mean (SD)	22.9	(16.8)	36.7	(56.7)	0.52
Alb, g/L; mean (SD)	3.6	(0.3)	3.9	(0.4)	0.03*
Na, mEq/L; mean (SD)	136.6	(3.2)	136.7	(2.0)	0.90
CRP, mg/dL; mean (SD)	7.3	(3.7)	3.8	(3.7)	0.02*

*SD* standard deviation, *KD* Kawasaki disease, *statistically significant defined as *p* <0.05

The detailed characteristics of the iKD group are shown in Table 3. Only 1 fulfilled the diagnostic criteria for KD, while the remaining 7 received the diagnosis of incomplete KD, and none of them met the AHA criteria of IVIG indication for incomplete KD. Seven of 8 iKD cases who received IVIG between 10–29 days after onset of illness showed either recurrence of fever or re-elevation of CRP before IVIG administration. However, the number of persistent KD symptoms, if present at all, was smaller than at the time of diagnosis. IVIG therapy led to the prompt reduction of CRP and cessation of recurrent fever in all 7 patients. All iKD cases initially showed normal echocardiogram results in terms of Japanese Ministry of Health’s CAL criteria and the z score, while 6 of them developed CAL between Day 11 and 23 (median 16) of illness. All of these patients showed a regression of the CALs, except for 1 patient with a giant aneurysm of the right coronary artery that partially regressed from 8.2 mm (z score 16.0)–6.2 mm (z score 10.9) in diameter at the time of the last follow-up (follow-up period: 6 months).

**Table 3:** Characteristics of the indolent KD

Case	Age, months	Sex	Initial symptoms		Recurrent fever, DOI	Re-elevated CRP, DOI	Persistent KD criteria	CAL, DOI	IVIG, DOI	Maximum CAL, mm (z score)	Prognosis
			Fever, days	KD criteria							
#1	34	M	5	4/5	day 12	N/A	2/5	day 14	day 15	#1: 4.9 (7.9)	Reg. in 10 months
#2	15	F	5	2/5	day 14	day14	0/5	day 18	day 18	#1: 3.8 (5.3)	Reg. in 1.5 months
#3	3	M	3	3/5	N/A	day11	0/5	N/A	day 29	#6: 1.9 (1.2)^a^	No complication
#4	6	F	4	3/5	N/A	day 11	0/5	day 11	day 11	#6: 2.9 (4.1)	Reg. in 1 week
#5	2	F	4	2/5	day 11	day 15	0/5	day 23	day 24	#6: 2.5 (2.9)	Reg. in 2 months
#6	20	F	4	3/5	day 9	N/A	2/5	N/A	day 10	N/A	No complication
#7	45	M	7	2/5	day 12	N/A	1/5	day 14	day 14	#1: 8.2 (16)	PR in 6 months
#8	5	F	5	3/5	day 12-18	N/A	0/5	day 19	N/A	#6: 2.7 (3.7)	Reg. in 1 months

*KD* Kawasaki disease, *IVIG* immunoglobulin, *CAL* coronary aretery lesion, *DOI* Days of illness, *N/A* not applicable, *Reg* regression, *PR* partially regressed ^a^It showed gradual dilatation from the initial measurement

## Discussion

In this retrospective cohort study of spontaneously defervesced KD patients, we found that there was no difference in the CAL complication rate between the conventional KD patients, who received initial IVIG therapy, and the dKD patients, who experienced spontaneous defervescence within 7 days of onset of illness without IVIG therapy and remained afebrile for at least 3 days. More than 10 % of these dKD patients consisted of iKD cases which later developed CALs or received late IVIG for ongoing, systemic inflammation after 10 days of illness. To our knowledge, this is the first cohort study to identify the characteristics of spontaneously defervesced KD and to elaborate the new disease concept of “indolent KD”.

Among our patient cohort, the CAL complication rate ≤ 30 days of illness in the dKD group was not significantly different from that of the tKD group who had received initial IVIG therapy (9.9 % vs. 7.6 %). In addition, these rates were comparable to those seen in the recent, nationwide surveys in Japan (10.0 % in 2007–2008 and 8.5 % in 2009–2010) [11, 12], and lower than those seen in 20–40 % of the historical cohort that received aspirin alone [13, 14]. These findings partially support the recommendations of the Japanese rather than American guidelines for KD management; the former state that IVIG therapy is not definitely indicated for spontaneously defervesced KD because of the lesser severity of the disease [1, 2]. However, the outcomes for the dKD group might have been better had initial IVIG therapy been administered. A risk-benefit assessment of IVIG therapy carefully weighing the possibility of preventing CALs against the problems accompanied by IVIG use is imperative for cases of spontaneously defervesced KD. None of the 7 iKD cases with an incomplete KD diagnosis observed in this study initially met the laboratory criteria from AHA, suggesting that caution be exercised in applying the AHA’s management strategy to cases of indolent KD.

In this study, approximately 10 % of the dKD cases were classified into the iKD group: those who experienced an indolent clinical course after 10 days of illness. To our knowledge, this is the first report describing this new sub-population of KD. Persistence, rather than recurrence, of the inflammation was assumed to account for its indolent course, considering all of the iKD cases experienced recurrence or continuous milder signs and symptoms of systemic inflammatory state after defervescence. The primary goal of KD management is the reduction of inflammation and fever before the eighth or ninth day of illness. For this purpose, IVIG should be administered by the seventh day post onset [2]. Although the dKD cases in our study apparently attained this outcome without IVIG, the iKD cases later developed CALs. All of the 8 iKD patients showed either recurrence of fever or re-elevation of CRP after spontaneous defervescence, while most of the initial KD symptoms subsided at the time of IVIG administration. Both fever and CRP elevation are well-known markers of ongoing inflammation in KD [15–17]. Our results indicate that clinical symptoms of KD besides fever are less useful for identifying sustained inflammation after spontaneous defervescence.

All of the iKD cases in this study experienced an immediate decrease in CRP levels and recurrent fever after IVIG administration. Moreover, improvement in the CALs was observed after late IVIG administration in all of the iKD cases presenting CALs with the exception of 1 case presenting a giant aneurysm. Although the efficacy of late IVIG therapy after 10 days of illness has not been established, some studies have reported on the benefits of suppressing ongoing systemic inflammation and ameliorating existing CALs [18, 19]. Thus we suggest that serial echocardiograms be conducted for spontaneous defervesced KD patients who present signs of sustained, systemic inflammation, and that IVIG be administered if CALs are detected.

This iKD cohort included a 3-year-old boy who developed giant aneurysms [20]. He presented to our ED with a fever of 6 days’ duration (37.6 C° at the time of presentation), bilateral conjunctivitis, and redness on the lips. He was monitored daily at the ED until Day 8 of illness when the fever spontaneously resolved. The patient experienced a single, temporary spike in his fever to >38 C°on Day 7 while at home, but was afebrile at the ED. His work-up showed WBC of 17,500/micro L, CRP of 7.9 mg/dL, and normal echocardiogram results on Day 7. He experienced mild, intermittent spikes of up to 37.7 C° until his next scheduled visit on Day 14 of illness where the coronary aneurysms were detected (#1: 7.7 mm, #5: 3.7 mm, #6: 5.9 mm). Prompt IVIG initiation on the occurrence of fever on Day 7 or more frequent monitoring of CRP followed by an echocardiogram could have prevented formation of the giant aneurysm.

CAL developments in the iKD patients were first detected during the second and third weeks of illness among the iKD patients except in the case of a 2-month-old girl when it was detected on Day 23. She presented with 4 days of fever accompanied by a mild rash, mild conjunctivitis, and mild redness of the soles. She was hospitalized and treated with antibiotics for possible infant sepsis, but experienced recurred, transient, episodic fever while presenting continuously elevated CRP levels. Serial echocardiograms performed to assess cardiac function and to or rule out of KD and endocarditis revealed worsening coronary artery dilatation, with the z score of #6 for Day 3, 8, 14, and 23 at 0.9 (1.7 mm), 0.9 (1.7 mm), 2.1 (2.2 mm), and 2.9 (2.5 mm), respectively. Her extremely young age and the mildness of the KD symptoms rendered prompt diagnosis difficult. However, a clear tendency towards CAL development was observed on Day 14 as with the other iKD cases.

The results of this study suggest that defervesced KD patients can be managed appropriately without IVIG administration under certain conditions. However, the following guidelines should be noted. First, prompt initiation of IVIG is recommended in all defervesced KD cases presenting a CAL or tendency to coronary artery dilation during the acute inflammatory stage. Second, as a higher CRP level may be the only sign at diagnosis predictive of an indolent course, IVIG should be more aggressively considered for those cases with higher CRP levels. Third, if withholding of immediate IVIG treatment is decided, caregivers should be advised to monitor temperature frequently (≥3 times /day) while the patient is at home. Should the axillary temperature rise to ≥ 37.5 C° (core temperature ≥ 38.0 C°), the patient should be taken to hospital immediately. Fourth, performing a weekly echocardiogram and follow-up of the CRP level are desirable until the third week of illness. More frequent monitorng should be considered in the presence of a recurrent fever. Fifth, IVIG administration should be considered for defervesced KD patients experiencing recurrent fever or re-elevation of CRP in the absence of CALs.

Based on the results of this study, we hypothesize that a CAL in KD develops when the product of the severity of inflammation and duration of inflammation exceeds a certain threshold for CAL, expressed as follows: CALth > S × D (CALth = threshold for a CAL formation, S = severity of inflammation, D = duration of inflammation). The lengthy duration of inflammation, manifested as a fever lasting multiple days, was previously reported as a useful marker of CAL development [15]. In this study, CALs developed in the iKD cases [median 16, range 11–23 (days of illness)] later than 8 to 10 days post disease onset as seen in conventional cases of CAL [2, 21]. The iKD can be considered to be a form of KD with low but persistent inflammation, since the fever initially subsides without IVIG. Our study found that a less severe form of the disease can nonetheless develop CALs after a longer period of inflammation. Hence delayed CAL development is possibly explained by the longer duration of inflammation despite its mildness.

Our study has several limitations. The retrospective nature of this study precluded elimination of several selection biases. First, other self-limiting, febrile, exanthematous childhood diseases could have been misdiagnosed as incomplete KD with spontaneous defervescence. However, all of the diagnoses of KD in our study were confirmed by at least 1 board-certified pediatrician in Japan with extensive experience diagnosing KD. Various corroborative findings in addition to the 5 cardinal KD symptoms were useful for diagnosis, such as the induration of the BCG inoculation site [22, 23]. Second, a milder form of spontaneously defervesced KD might not have been identified. This is an innate limitation of clinical studies of KD, which depend on an unreliable clinical diagnosis. Third, the extremely high prevalence of CALs among iKD cases is possibly explained by a certain sub-population of dKD patients who presented severe recurrent fever necessitating laboratory tests or echocardiograms. Fourth, there was no protocol for monitoring dKD, other than the instruction to re-visit the hospitals if fever or KD symptoms recur, and to perform an echocardiogram at 1 month after diagnosis. Other aspects of managements were determined at the discretion of clinicians, such as the timing of laboratory tests, echocardiogram, and clinic visits. Failure to detect CRP elevation or minor CALs was a possibility. However, an echocardiogram performed at 1 month post onset assured detection of all major acute CALs. The CAL was defined according to the Japanese Ministry of Health criteria because a number of cases lacked the information necessary for the calculation of the z score, possibly resulting in the under-detection of true CALs [24]. However, none of the iKD patients showed a significantly increased z score for coronary artery diameter at the initial echocardiogram.

## Conclusion

In conclusion, we showed that spontaneously defervesced KD without initial IVIG included a relatively benign group of patients, who can be managed without prompt IVIG treatment as long as they were monitored closely. Caution should be taken in regard to indolent KD cases since approximately 10 % of the defervesced cases that later develop CALs. Timely initiation of IVIG treatment based on close monitoring of recurrent fever and re-elevation of CRP supplemented by an echocardiogram is important for effective management of indolent KD.

## Abbreviations

AHA: American Heart Association
CAL: coronary artery lesion
dKD: defervesced kawasaki disease
iKD: indolent kawasaki disease
IVIG: intravenous immunoglobulin
KD: kawasaki disease
rKD: resolved kawasaki disease
tKD: treated kawasaki disease
